# A Hierarchical Multi-Task Learning Framework for Semantic Annotation in Tabular Data

**DOI:** 10.3390/e26080664

**Published:** 2024-08-04

**Authors:** Jie Wu, Mengshu Hou

**Affiliations:** School of Computer Science and Engineering, University of Electronic Science and Technology of China, Chengdu 611731, China; jiewu@std.uestc.edu.cn

**Keywords:** tabular data, natural language processing, table semantic annotation, table interpretation, multi-task learning

## Abstract

To optimize the utilization and analysis of tables, it is essential to recognize and understand their semantics comprehensively. This requirement is especially critical given that many tables lack explicit annotations, necessitating the identification of column types and inter-column relationships. Such identification can significantly augment data quality, streamline data integration, and support data analysis and mining. Current table annotation models often address each subtask independently, which may result in the neglect of constraints and contextual information, causing relational ambiguities and inference errors. To address this issue, we propose a unified multi-task learning framework capable of concurrently handling multiple tasks within a single model, including column named entity recognition, column type identification, and inter-column relationship detection. By integrating these tasks, the framework exploits their interrelations, facilitating the exchange of shallow features and the sharing of representations. Their cooperation enables each task to leverage insights from the others, thereby improving the performance of individual subtasks and enhancing the model’s overall generalization capabilities. Notably, our model is designed to employ only the internal information of tabular data, avoiding reliance on external context or knowledge graphs. This design ensures robust performance even with limited input information. Extensive experiments demonstrate the superior performance of our model across various tasks, validating the effectiveness of unified multi-task learning framework in the recognition and comprehension of table semantics.

## 1. Introduction

Tables are employed in a wide variety of applications, from simple personal records to complex enterprise data management systems, making them one of the most prevalent and important data formats in documents, web pages, and databases. Regardless of whether the data are textual, numerical, or boolean, tables are adept at presenting information in a compact and clear format, thus supporting efficient storage, retrieval, and analysis. These attributes confer significant advantages to tables in data management, ensuring that data remains organized and accessible, and allowing for effective data cleaning, aggregation, and searching [1]. In addition, tables provide a rich and structured source of information for various tasks, including question-answering systems, information extraction, knowledge graph construction, and fact verification [2,3]. The absence of essential metadata, such as column names and relationships, considerably complicates these tasks, especially for web-sourced tables that often lack proper annotations. To exploit the full potential of tables, it is crucial to accurately interpret the semantics of the columns and their interrelations. This underscores the necessity for precise table annotation, which augments readability and usability, establishing a robust foundation for various purposes of tables.

Table semantic annotation, also known as table semantic interpretation, refers to the assignment of semantic tags to elements of a table. Advancements in table annotation primarily follow two principal directions: those based on knowledge graph methodologies, and those rooted in deep learning paradigms. Early research mainly utilized look-up-based methods [4,5,6], relying on real-time queries to knowledge bases (e.g., DBpedia and Wikidata) and keyword matching within cell entities, or leveraging relational structures and contextual information from knowledge bases for annotation. Regrettably, the coverage of knowledge bases often falls short of fully supporting all entities and relationships present within tables. Inspired by the efficacy exhibited by pre-trained language models (PLMs), recent years have seen the emergence of pre-trained models specifically tailored for tabular data [7,8]. These models generally adopt self-supervised learning paradigm of PLMs. Nevertheless, the unique nature of data cells within tables, lacking the strong interconnection properties of textual or graphical data, resulting in models that, even after extensive pre-training on large-scale tabular corpus, still fall short of the generalization capabilities of PLMs. Despite significant progress having been made within table semantic annotation in recent years, several challenges remain. The primary issue is the high computational resource demand. Both query-based and pre-training methods necessitate significant computational resources, particularly evident in tabular pre-training due to the processing massive data. Another challenge is the limitation on table length. Existing models impose restrictions on input data length, potentially leading to truncation of table content and loss of critical information. Furthermore, current table annotation models typically focus solely to the detection of column types, neglecting inter-column dependencies, which needs further academic inquiry and technique refinement.

**Figure 1:**
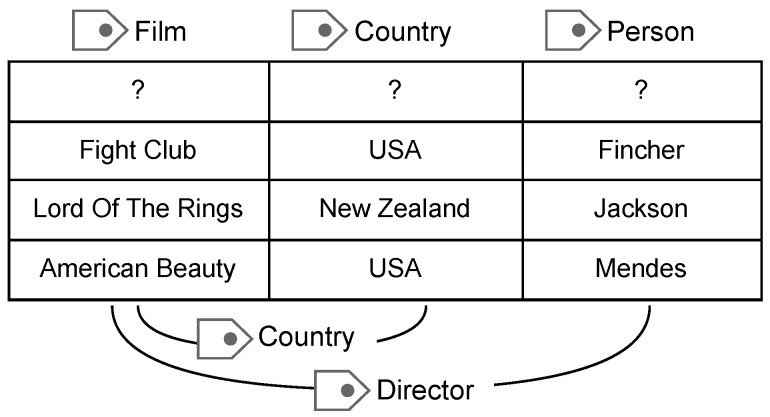
An example of table semantic annotation. The goal is to assign semantic tags to columns and column pairs within the table.

To confront aforementioned issues, we prioritize task correlations while simultaneously addressing other relevant concerns. In tabular data, it is customary for cells within one column to pertain to a consistent semantic type, while different columns collectively contribute to the overarching theme of one table. Figure 1 presents a headerless table, which requires annotation of column types and their relationships. The exemplified table’s theme is “film”, with columns representing film titles, belonging countries, and directors, delineating distinct yet interconnected concepts. Identifying column types depends on recognizing commonalities among values within each column, with additional contextual cues potentially derived from the values and types of other columns. This principle also applies to discerning relationships between columns. For instance, distinguishing the first column as “film” narrows possible types for the third column, making it unlikely to represent “engineer” or “player”. Therefore, a comprehensive understanding of the table’s semantics necessitates consideration of its entire context. Column types and inter-column relationships are intricately intertwined, exhibiting many shared features. Identifying column types can help ascertain inter-column relationships, and vice versa, discerned relationships can provide insights conducive to determining column types. However, existing table annotation methods typically operate within a single task, ignoring intrinsic interdependencies among tasks, leading to suboptimal information extraction and misalignment with the thematic coherence. To address this issue and exploit the latent interaction between subtasks, we propose an innovative model based on joint multi-task learning, unifying various annotation tasks within one framework. It initially linearizes the entire table by columns, and then concatenates them into encoder to generate contextual representations for each column. Subsequently, the model undergoes iterative training cycles, during which supervision signals from diverse subtasks are alternately integrated. In addition to primary tasks of identifying column types and inter-column relationships, we incorporate a basic yet valuable task of Named Entity Recognition (NER) within columns. Recognizing named entities in columns can provide foundational entity information and has been proven to be efficient in subsequent experiments. Through multi-task learning, it fosters dynamic exchange of information across entities, column types, and inter-column relationships, resulting in more expressive data representations without the need for external knowledge bases or additional page content. Furthermore, this approach facilitates better data leveraging by model, consequently promoting both the overall generalization capacity and learning efficiency.

Our contributions are threefold:

- We introduce a novel representation learning framework based on multi-task learning for table semantic annotation. The central concept is the integration of diverse tasks into a unified learning architecture, leveraging inter-task correlations and domain knowledge among multiple table semantic annotation subtasks. This enables model to concurrently learn and understand both commonalities and differences among different subtasks, thereby augmenting performance of each individual subtask.
- Our model achieves remarkable performance without relying on external knowledge bases, linked web resources, or pre-trained tabular models, instead utilizing only target datasets. This not only reduces dependence on external resources, but also mitigates implementation costs and complexities, offering more flexibility and scalability.
- Extensive experimentation validates the superiority of our proposed model in multi-task learning and representation learning, as well as its advantages in data utilization and training efficiency.

## 2. Related Work

### 2.1. Table Semantic Annotation

Research on table semantic annotation primarily concentrates on predicting column types, a critical task in understanding and utilizing tabular data. Related work covers the development of advanced feature extraction techniques, the application of deep learning algorithms, and the integration of external knowledge graphs (KGs). These feature-based approaches capture the semantics embedded within tables through extraction of manual, statistical, and semantic features. For example, SemanticTyper [9] uses TF-IDF and statistical test methods to extract semantic features from tables. Sherlock [10] refines feature extraction by incorporating character distributions, semantic embeddings, and global statistics at multiple levels of granularity. Building upon Sherlock, Sato [11] introduces additional features, such as topic elements and contextual information, thereby improving the semantic interpretation of tables. Furthermore, some methods integrate external KGs to enrich their prediction frameworks with supplementary contextual clues, as demonstrated by systems like ColNet [5], Meimei [12], and $C^{2}$ [13]. With the rise of large language models (LLMs), some studies have emerged to apply LLMs for table understanding, but there has been relatively less work on using LLMs for table annotation tasks. Typical practices [14,15] include designing prompts to enable GPT-3.5/4 [16] to perform table annotation and conducting comparisons in zero-shot and few-shot scenarios. The above advancements in table semantic annotation have made substantial progress in the ability to effectively interpret and utilize tabular data, providing some advantages in extracting and understanding complex semantic information.

### 2.2. Pre-Trained Tabular Models

Driven by notable successes in pre-training within natural language processing, there has been a growing interest in developing pre-training techniques specifically for tabular data in recent years. These efforts aim to foster a general understanding of tabular data by learning from extensive collections of tables using appropriate pre-training objectives, and subsequently deploying pre-trained model to various downstream tasks. Although tabular pre-training methods largely imitate the idea of text pre-training, they have not yet achieved satisfactory generalization and applicability. The dominant approach in tabular pre-training employs a transformer-based architecture, with progress mainly divided into two areas: architectural enhancements and training refinements. Architectural innovations primarily focus on incorporation of table’s structure into model, including techniques for encoding structural information at model’s input layer [8,17] or embedding such information within model’s internal mechanisms [7,18]. Improvements of training strategies predominantly explore novel pre-training objectives, such as masking, corrupting, and pairwise ranking [19,20,21].

### 2.3. Multi-Task Learning

Multi-task learning is a flexible and valuable learning paradigm that maximizes the use of shared information across tasks while simultaneously handling multiple related tasks. Compared to single-task learning, multi-task learning offers several advantages, such as improved data utilization, enhanced inference efficiency, and better generalization. It is widely applied in various fields, including natural language processing, computer vision, recommendation systems, and robotics. The remarkable success of deep MTL can be attributed to its ability to extract rich representations and share valid information. According to the taxonomy by Ruder [22], multi-task sharing can be divided into two categories: hard sharing like uniform-layer architecture, and soft sharing like coupled- and shared-layer architectures. Hard sharing requires tasks to have the same parameters in the shallow layer, while soft sharing encourages each task to maintain its own shallow parameters in which the features of related tasks are interactively propagated, including aggregation, fusion, attention [23,24], etc. Beyond direct information sharing on encoder or decoder, there are other forms of sharing, such as adapter [25,26] and hypernetwork [27,28]. The utilization of task relatedness is important for multi-task learning, which is reflected in the construction of model architecture. The parallel architecture [29,30] is the most commonly used multi-task model structure, and there are no dependencies between tasks other than sharing in middle layers. The hierarchical architecture [31,32] considers the hierarchical relationship between tasks, where the features or outputs of one task are used as inputs to other tasks. As neural networks continue to grow in scale, tasks can benefit even more from multi-task learning.

## 3. Notations and Problem Definition

To increase clarity and simplify referencing, we summarize the majority of significant symbols in Table 1. The tables discussed herein primarily pertain to relational tables characterized by variable row and column counts. Each table $\left\{c_{i,r}|i\in \left(1,m\right),r\in \left(1,n\right)\right\}$ contains *m* rows and *n* columns with the content $c_{i,r}$ in the (i,r)-th cell. The value $c_{i,r}$ could be a number, a word or a phrase. Notably, the tasks addressed in this study are all defined along the dimension of table columns, as described below:

- Problem 1: Column named entity recognition (NER). Given a table T (without table headers or contextual information) and a set of simple named entity types $C_{ner}$, the goal is to predict the entity type of target column *i*, which can best describe most of the entities in column. This process is denoted as $p(C_{ner}|T,i)$.
- Problem 2: Column type annotation (CTA). Given a table T (without table headers or contextual information) and a set of column semantic types $C_{cta}$, the goal is to predict the semantic type of target column *i* based on only the table content. This process is represented as $p(C_{cta}|T,i)$.
- Problem 3: Inter-column relationship annotation (CRA). Given a table T (without table headers or contextual information) and a set of inter-column relationship types $C_{cra}$, the goal is to predict the relation semantic type of target column pair (i,j) based solely on the table content. This process can be represented as $p(C_{cra}|T,i,j)$.

**Table 1:** Notations with corresponding descriptions.

Notations	Description
T	Standard relational table without headers
$C_{i}$	The *i*-th column in table T
$c_{i,r}$	the value of cell at the *i*-th column, *r*-th row of table
$C_{cta}$	The set of all column semantic types
$C_{cra}$	The set of all inter-column relationship types
$C_{ner}$	The set of all column named entity types
$e_{i}$	Contextual representation of *i*-th column in encoding layer
$h_{i}$,$h_{ij}$	Output vector of *i*-th column or (i,j)-th column-pair in task layer
$p_{i}$,$p_{ij}$	Prediction probability of *i*-th column or (i,j)-th column-pair in task layer

Our proposed framework includes three typical table-related tasks: column named entity recognition, column type annotation, inter-column relationship annotation. The target for the first two tasks is semantics of a single column, while the target for the latter is semantics of a column pair.

## 4. Methodology

**Figure 2:**
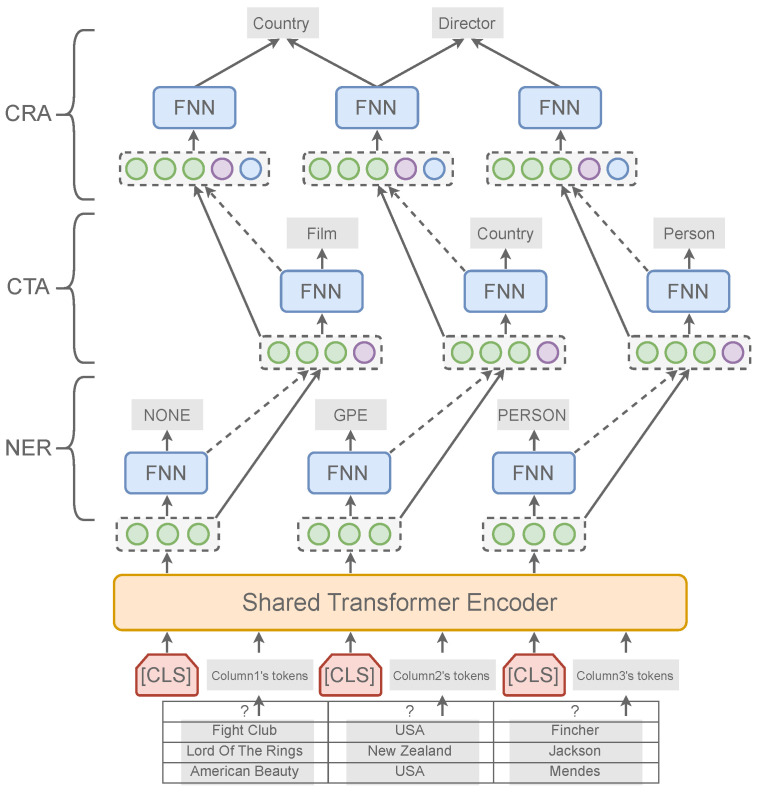
The architecture of our multi-task learning framework. Our model features a shared bottom encoder coupled with multiple associated classifiers. Once the table, serialized into text, is encoded, the representations of all columns are forwarded to the upper classifiers. Information is then processed sequentially according to the task hierarchy of column named entity recognition (NER), column type annotation (CTA), and inter-column relationship annotation (CRA).

Figure 2 illustrates the architecture of our model, which consists of a shared encoder based on a transformer structure and three upper-level classifiers for related tasks. In the following subsections, an in-depth explanation of the methodology will be provided. Section 4.1 describes the way how to serialize table and represent columns. Section 4.2, Section 4.3 and Section 4.4, respectively, introduce the implementation of three subtasks in our multi-task learning model, including column named entity recognition (NER), column type annotation (CTA), inter-column relationship annotation (CRA). Section 4.5 presents the multi-task training strategy.

### 4.1. Table Serialization and Column Representations

We adapt Transformer as our encoder, which is a deep learning model based on self-attention mechanism [33] and excels at efficiently capturing contextual semantics. To conform to Transformer’s input requirements, we need to linearize the table, transforming it from a two-dimensional structure into a one-dimensional sequence. We make the whole table as one complete input to the encoder, rather than just a single column or a few columns. This allows the encoder to perceive global contextual information of entire table when generating representation of each column. For an m×n table T with cells $\left\{c_{i,r}|i\in \left(1,m\right),r\in \left(1,n\right)\right\}$, where *i* and *r* represent column and row indices, respectively, we perform a vertical scan in column order, sequentially concatenating the contents of each column’s cells. To distinguish different columns, we place a special [CLS] token at the start of each column and append a [SEP] token at its end. In this way, we obtain the linearized column sequences and table sequence, represented by $C_{i}^{*}$ and $T^{*}$, respectively:

(1) $$C_{i}^{*}=\left[\mathrm{CLS}\right]c_{i,1}\ldots c_{i,n}\left[\mathrm{SEP}\right],$$

(2) $$T^{*}=\left[\mathrm{CLS}\right]c_{1,1}\ldots c_{1,n}\left[\mathrm{CLS}\right]\ldots \left[\mathrm{CLS}\right]c_{m,1}\ldots c_{m,n}\left[\mathrm{SEP}\right].$$

Although the [CLS] and [SEP] tokens themselves do not possess specific semantic meanings, their usage has become a de facto standard in PLMs. In the table annotation task, under the guidance of multi-task learning supervision signals, the knowledge and extracted features of various subtasks are distilled and aggregated into the [CLS] token. Consequently, the hidden vector of the [CLS] token can thus be regarded as representing the key features and semantic information of corresponding column sequence:

(3) $$e_{i}=\mathrm{encoder}\left(T^{*},i\right),$$

where $e_{i}\in R^{d}$ is the contextual representation vector for *i*-th column in T and *d* is the dimension of hidden vector in Transformer.

### 4.2. Column Named Entity Recognition

We design an additional classification task, referred to as column named entity recognition (NER), which aims to predict the named entity types corresponding to table columns, thereby incorporating foundational named entity information. Despite the relatively limited number of entity types and the simplicity of this task, it serves as a foundation for more complex column semantic annotation tasks, offering features and guidance that strengthen model’s performance in these advanced tasks. This task is strategically positioned at the base layer of our framework. The hidden vectors derived from [CLS] tokens by Transformer are directly input into the classifier within NER layer:

(4) $$h_{i}^{ner}=\tanh\left(e_{i}W_{1}^{ner}\right)W_{2}^{ner},$$

(5) $$p_{i}^{ner}=\mathrm{softmax}\left(h_{i}^{ner}\right),$$

where $W_{1}^{ner}\in R^{d\times d}$ and $W_{2}^{ner}\in R^{d\times n_{ner}}$ are learnable parameters of the feed-forward network, $h_{i}^{ner}$ is the output vector of dense layer for column NER, $p_{i}^{ner}\in R^{n_{ner}}$ is the probabilities of column NER type for *i*-th column, and $n_{ner}$ is type number of column NER. The cross-entropy loss $L_{\mathrm{ner}}$ is calculated for classification in NER during training.

### 4.3. Column Type Annotation

Column type annotation (CTA) aims to predict the semantic types of columns in a table. CTA is another task focused on table columns, but with a more diverse and complex set of classification categories. The CTA layer is positioned above both the encoder and NER layer. It simultaneously receives output vectors from encoder and intermediate vectors from NER layer, which are concatenated to serve as input for predicting column types. This method incorporates NER features into the CTA process, thereby infusing additional NER knowledge to increase accuracy of column type annotation. It enriches the information available for CTA task, which can be delineated as follows:

(6) $$h_{i}^{cta}=\tanh\left(\left(e_{i}⊕h_{i}^{ner}\right)W_{1}^{cta}\right)W_{2}^{cta},$$

(7) $$p_{i}^{cta}=\mathrm{softmax}\left(h_{i}^{cta}\right),$$

where ⊕ is the concatenate operation between vectors, $W_{1}^{cta}\in R^{(d+n_{ner})\times d}$ and $W_{2}^{cta}\in R^{d\times n_{cta}}$ are learnable parameters of the feed-forward network in CTA layer, $h_{i}^{cta}$ is the output vector of dense layer for column type prediction, $p_{i}^{cta}\in R^{n_{cta}}$ is the probabilities of column type for *i*-th column, and $n_{cta}$ is the number of column types. The objective loss $L_{\mathrm{cta}}$ for column type annotation is cross-entropy loss.

### 4.4. Inter-Column Relationship Annotation

Inter-column relationship annotation (CRA) focuses on predicting relationships between two columns within a table. Due to the complexity of relationship categories, this classification task is particularly challenging. Therefore, in our design, CRA is positioned at the highest layer. Incorporating semantic information from column types into CRA classifier aids in a deeper understanding of each column’s meaning, thereby improving the accuracy of inter-column relationship annotation. The CRA layer includes a classifier situated above CTA layer. It simultaneously receives two columns’ output vectors from Transformer, along with intermediate vectors from CTA layer, as input. Since the intermediate vectors from CTA layer already encompass NER knowledge, there is no need to reintroduce vectors from NER layer at this stage. This process can be delineated as follows:

(8) $$h_{ij}^{cra}=\tanh\left(\left(e_{i}⊕h_{i}^{cta}⊕e_{j}⊕h_{j}^{cta}\right)W_{1}^{cra}\right)W_{2}^{cra},$$

(9) $$p_{ij}^{cra}=\mathrm{softmax}\left(h_{ij}^{cra}\right),$$

where $W_{1}^{cra}\in R^{2(d+n_{cta})\times d}$ and $W_{2}^{cra}\in R^{d\times n_{cra}}$ are learnable parameters of the feed-forward network in CRA layer, $h_{ij}^{cra}$ is the output vector of dense layer for inter-column relationship prediction, $p_{ij}^{cra}\in R^{n_{cra}}$ is the probabilities of column type between *i*-th and *j*-th columns, and $n_{cra}$ is the number of column relations. The objective loss $L_{\mathrm{cra}}$ for inter-column relationship annotation is cross-entropy loss.

### 4.5. Joint Multi-Task Learning

Despite some research suggesting that employing a concatenated form of multi-task learning might degrade model performance when task correlations are weak, our model benefits from strong inter-task correlations and a suitable task difficulty hierarchy. Hence, adopting this method is justifiable, and subsequent experimental results validate its effectiveness. Considering the discrepancies in sample distribution among three tasks, simultaneous training risks data leakage issues, necessitating either replication or deletion of data. Consequently, we choose the cyclic training strategy where each epoch alternates one task’s full training before proceeding to next task. This optimizes data utilization and accommodates imbalanced task data volumes, and avoids selection of weighted hyper-parameters for task loss functions. The complete training strategy for joint multi-task learning is outlined in Algorithm 1.
**Algorithm 1:** Training strategy of our multi-task learning framework.
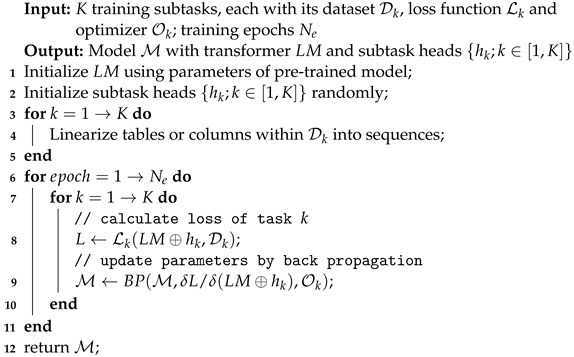


## 5. Experiments

### 5.1. Datasets

We conduct experiments on two datasets, SemTab2019 [34] and HardTables2022 [35], which include annotations for both column types and inter-column relationships. Both datasets contain vertical relational web tables from Wikipedia with semantic annotations at various levels of detail. The average number of rows in tables from HardTables2022 is much smaller than that in SemTab2019, making annotation more challenging. Table 2 presents a summary of basic statistics of two datasets. Due to the absence of a manually annotated dataset for column NER task, we use the NER annotation tool spaCy [36] to identify entity types of all cells in each column, assigning the most frequent entity type as the column entity type. In accordance with prior research practices, all table headers are excluded. With no overlap among tables, 10% of the samples are randomly chosen as test set, while the remaining data are divided into five folds for cross-validation.

To evaluate model performance, we employ two types of F1 score metrics: micro-average F1 score and macro-average F1 score. The micro-average F1 score considers all instances across classes to compute an overall average, emphasizing performance on common classes. Conversely, the macro-average F1 score treats all classes equally by averaging F1 scores of each class, providing a balanced view of performance across all classes, regardless of their frequencies.

### 5.2. Experimental Settings

The implementation of our model is based on Pytorch and transformer library [37,38]. The parameter initialization for encoder Equation (3) is derived from Roberta-base [39], which is a BERT-like transformer consisting of 12 layers. The hidden vector’s dimension *d* is set to 768. We train models for 40 epochs on each fold of dataset. The AdamW optimizer is employed with learning rates of 1 × 10^−5^, 3 × 10^−5^ and 4 × 10^−5^ for NER, CTA and CRA, respectively. All experiments are conducted on a single RTX-Titan GPU with a batch size of 16.

### 5.3. Results and Analysis

**Overall performance.** Our model is compared with competitive baselines, including TaBERT [7], TABBIE [8], DODUO [40]. The overall comparison with baseline models is outlined in Table 3 and Table 4, which is based on average performance of 5-fold tests. The experimental results indicate that our model achieves outstanding performance, with two key metrics of 82.4%/63.6% and 72.6%/62.8% on Semtab2019 dataset, and 86.3%/73.2% and 61.5%/51.9% on HardTables2022 dataset, significantly outperforming the baseline models. This highlights the model’s robust feature extraction capabilities and its high adaptability to task requirements. Comparing the improvements in two F1 scores, our model shows valuable enhancement in macro F1, indicating that our multi-task learning framework can strengthen the learning ability for small sample categories. Furthermore, our model’s performance compared to DODUO suggests that its architecture is more apt for table annotation tasks, enabling more suitable alignment of inter-task correlations and realizing real task information interaction. Unlike many tabular understanding models, such as TaBERT and TABBIE, which require pre-training on extensive data, our model excels by employing a refined model design and a multi-task learning strategy, achieving exceptional results with training solely on target datasets. The collaborations between three tasks contribute to a substantial improvement of overall performance.

**Figure 3:**
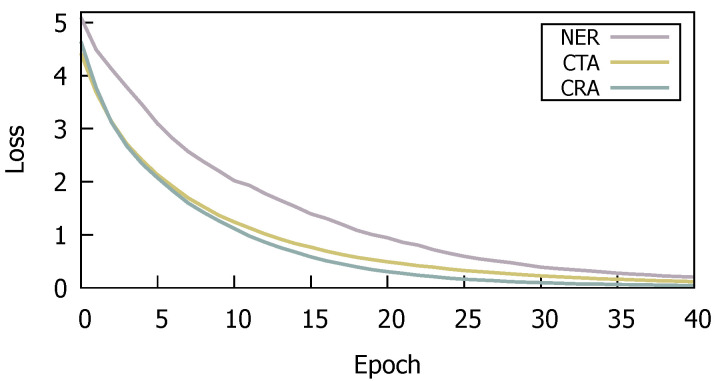
The training losses of three subtasks.

We illustrate the training losses on SemTab2019 during training process in Figure 3. Notably, the training losses for the primary tasks of CTA and CRA exhibit closely aligned descending trends, whereas the training loss for NER task displays a slightly slower decrease. The phenomenon can be attributed to our prescribed learning rate. This observation underscores the importance of flexibly adjusting training strategies during training process to accommodate varying task complexities and align with model’s requirements. Our training strategy, characterized by its simplicity and efficacy, ensures balanced process across diverse tasks. It allows for appropriate adjustments based on each task’s characteristics, thereby optimizing the training process, promoting overall model performance, and proficiently meeting the demands of complex real-world applications.

**Ablation analysis.** To analyze the distinct roles of different subtasks within our model, we conduct ablation experiments and present results in Table 3 and Table 4. The “target only” indicates training our model solely on a single target task, while the remaining three ablation experiments involve training with the exclusion of corresponding subtasks. On the Semtab2019 dataset, training solely on a single target task results in a performance decrease of 4.3%/10.4% and 2.9%/6.0% for CTA and CRA tasks, respectively, compared to full training. The introduction of NER task promotes both CTA and CRA tasks, proving the efficacy of incorporating entity information. Collaboration between CTA and CRA tasks is evident in their mutual performance enhancement, particularly in increasing macro average F1 scores. On HardTables2022 dataset, where most tables contain numerical data with fewer entities, the influence of NER is considerably reduced. Despite this, CTA continues to significantly benefit CRA. Additionally, notable augmentation in macro F1 compared to micro F1 highlights model’s proficiency in boosting classification accuracy for categories with fewer instances. These experimental results underscore the robust inter-task correlations successfully captured by our model design, which greatly reinforces overall performance.

**The number of input rows.** To investigate the impact of maximum number of input rows for tables, we conduct experiments on SemTab2019, which contains many large tables. As detailed in Table 5, “*n* row” denotes inputting only the first *n* rows of table, while “max length” refers to truncating table sequence according to model’s maximum input length. Experimental results indicate that our model maintains satisfactory performance, even when utilizing a reduced number of input rows, highlighting its capability to effectively comprehend and utilize input data. This adaptability underscores its suitability for scenarios involving small-scale tables.

**Figure 4:**
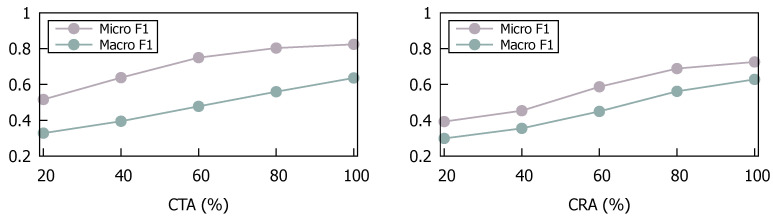
Performance of training under different proportions of dataset.

**The size of training data.** Figure 4 illustrates our model’s training outcomes across varying data volumes of SemTab2019, aimed at evaluating its learning efficacy with reduced training data. The training data for each class is uniformly and randomly partitioned into subsets of different sizes for training, while test set remains unchanged. Significantly, a notable decline in performance is observed when training data volume drops below 60%, primarily due to the skewed distribution of samples among categories within the dataset. However, training with 80% of the dataset resulted in only a marginal decrease in micro F1 scores for CTA and CRA tasks by 2.1% and 3.8%, respectively. These findings underscore model’s robustness in achieving relatively acceptable performance under conditions with limited yet balanced samples.

**Compared with weighted loss training.** During the training process of multi-task learning, we employ a cyclic strategy, sequentially training each task in each epoch. For comparison, we attempt another strategy, which is to train using weighted losses for multiple subtasks. In this scenario, training samples need to be simultaneously labeled for both CTA and CRA. However, not every sample in datasets meets this condition; some tables are only labeled for column types, while some are only labeled for inter-column relationships, such as HardTables2022 with approximately 12% of data being non-overlapping. For weighted loss training, we experiment with various weight combinations, among which the combination (0.1, 0.4, 0.5) has the best results, with a learning rate set at 2 × 10^−5^. The performance on HardTables2022 are 84.6%/68.4% and 57.2%/45.2%, lower than those achieved using our cyclic training strategy. This indicates that adopting an alternating training method for subtasks offers certain advantages in cases of task sample imbalance.

**Figure 5:**
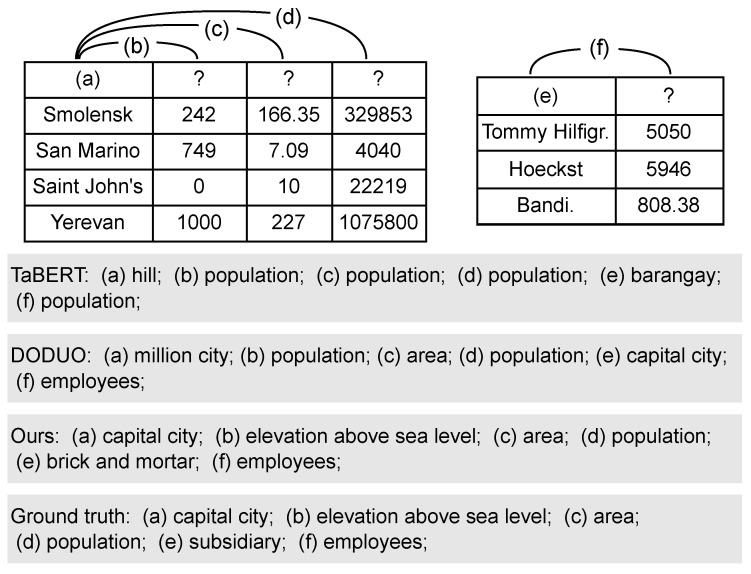
The case study on HardTables2022 dataset. It includes the prediction results of different models for column types and inter-column relationships.

**Case study.** To more intuitively show the effect of our model, we present a case study in Figure 5, comparing the results of two samples from HardTables2022 dataset. In the first case, it can be observed that TaBERT incorrectly predicts multiple columns and column relationships, where the types of purely numeric columns are difficult to distinguish. By utilizing information of NER and inter-column relationships, our model can accurately predict the relationship types of numerical columns. In the second failure case, all models are wrong in their predictions of column type (e). Specifically, TaBERT predicts “barangay”, DODUO predicts “capital city”, and our model predicts “brick and mortar”. Since “brick and mortar” and “subsidiary” are semantically similar, our model’s prediction is closer to the correct prediction. These cases demonstrate that when predicting a single column type or inter-column relationship is challenging, the interaction between column types and inter-column relationships becomes crucial. The effective annotations underscore the quality and utility of our model, validating its superiority in handling difficult annotation tasks.

## 6. Conclusions

This paper proposes a joint multi-task learning framework for table semantic annotation, designed to fully leverage the interrelations among three column-based tasks: column named entity recognition (NER), column type annotation (CTA), and inter-column relationship annotation (CRA). Current table annotation methods typically optimize for a single task, neglecting the potential interdependencies among tasks. Our approach addresses these limitations through information sharing and joint training. Specifically, our framework employs a shared encoder to generate general semantic representations of columns, which are subsequently processed by multiple specialized classifiers for three subtasks. This design allows each subtask to benefit not only from its own data but also from auxiliary information provided by related tasks, thus promoting overall performance. We conduct extensive experiments on public datasets to examine the mutual influences among subtasks, performance across varying data scales and lengths, and the efficacy of our model in terms of training efficiency and data utilization. Experimental results reveal the foundational contribution of NER task, significant mutual reinforcement between CTA and CRA tasks, and the model’s high training efficiency. Our research offers novel insights into table semantic annotation and robustly supports application of multi-task learning in practical scenarios. Future research will focus on further optimizing training process to enhance model’s efficiency and intelligence, exploring model’s applicability to additional tasks and knowledge domains, and expanding its generalization capabilities and application fields.

## Figures and Tables

**Table 2:** Basic statistics of datasets.

Dataset	CTA			CRA		
	# Type	# Sample	# Table	# Type	# Sample	# Table
SemTab2019	275	7614	3045	550	10,438	3025
HardTables2022	492	8072	6420	402	9772	6301

**Table 3:** Experimental results of different models on SemTab2019 dataset.

Model	CTA		CRA	
	Micro F1	Macro F1	Micro F1	Macro F1
TaBERT [7]	0.661 ± 0.012	0.412 ± 0.017	0.561 ± 0.016	0.440 ± 0.019
TABBIE [8]	0.774 ± 0.010	0.607 ± 0.011	0.673 ± 0.014	0.572 ± 0.010
DODUO [40]	0.795 ± 0.011	0.583 ± 0.013	0.690 ± 0.010	0.573 ± 0.014
Ours	0.824 ± 0.009	0.636 ± 0.012	0.726 ± 0.011	0.628 ± 0.013
w/ target only	0.781 ± 0.010	0.532 ± 0.015	0.697 ± 0.008	0.568 ± 0.011
w/o NER	0.808 ± 0.011	0.599 ± 0.009	0.716 ± 0.010	0.604 ± 0.013
w/o CTA	-	-	0.710 ± 0.008	0.584 ± 0.013
w/o CRA	0.798 ± 0.012	0.566 ± 0.014	-	-

**Table 4:** Experimental results of different models on HardTables2022 dataset.

Model	CTA		CRA	
	Micro F1	Macro F1	Micro F1	Macro F1
TaBERT [7]	0.684 ± 0.011	0.466 ± 0.014	0.429 ± 0.012	0.325 ± 0.016
TABBIE [8]	0.822 ± 0.010	0.683 ± 0.010	0.551 ± 0.014	0.488 ± 0.011
DODUO [40]	0.846 ± 0.011	0.689 ± 0.012	0.569 ± 0.011	0.476 ± 0.013
Ours	0.863 ± 0.010	0.732 ± 0.009	0.615 ± 0.009	0.519 ± 0.011
w/target only	0.845 ± 0.008	0.711 ± 0.010	0.549 ± 0.012	0.462 ± 0.012
w/o NER	0.860 ± 0.006	0.727 ± 0.011	0.613 ± 0.013	0.516 ± 0.010
w/o CTA	-	-	0.556 ± 0.011	0.473 ± 0.015
w/o CRA	0.850 ± 0.008	0.720 ± 0.010	-	-

**Table 5:** Performance of training at different input lengths.

Max Rows	CTA		CRA	
	Micro avg F1	Macro avg F1	Micro avg F1	Macro avg F1
2 rows	0.484 ± 0.006	0.338 ± 0.008	0.416 ± 0.008	0.343 ± 0.009
4 rows	0.755 ± 0.009	0.518 ± 0.011	0.670 ± 0.012	0.572 ± 0.015
8 rows	0.774 ± 0.011	0.584 ± 0.013	0.694 ± 0.008	0.592 ± 0.012
16 rows	0.809 ± 0.009	0.610 ± 0.010	0.704 ± 0.011	0.605 ± 0.012
max length	0.824 ± 0.009	0.636 ± 0.012	0.726 ± 0.011	0.628 ± 0.013

## Data Availability

All data that support the findings of this study are included within the paper.
